# Tuberculosis in Poland: Epidemiological and Molecular Analysis during the COVID-19 Pandemic

**DOI:** 10.3390/diagnostics12081883

**Published:** 2022-08-03

**Authors:** Dagmara Borkowska-Tatar, Anna Zabost, Monika Kozińska, Ewa Augustynowicz-Kopeć

**Affiliations:** Department of Microbiology, National Tuberculosis and Lung Diseases Research Institute, 01-138 Warsaw, Poland; a.zabost@igichp.edu.pl (A.Z.); m.kozinska@igichp.edu.pl (M.K.); e.kopec@igichp.edu.pl (E.A.-K.)

**Keywords:** tuberculosis, COVID-19 pandemic, drug resistance, spoligotyping, Poland

## Abstract

The COVID-19 pandemic may have a negative impact on the proper implementation of TB control programmes and may increase TB incidence rates in the near future. The aim of this study was to perform an epidemiological and molecular analysis of *Mycobacterium tuberculosis* strains cultured from tuberculosis patients in Poland in 2020 and to compare the results of monitoring drug-resistant tuberculosis in Poland with previous studies in 2012 and 2016. The analysis was based on questionnaires and strains sent by regional laboratories during the 12 months of 2020. Molecular analysis was performed by spoligotyping 20% of the strains sensitive to the four primary antimycobacterial drugs and all of the drug-resistant strains. The number of strains sent for analysis dropped threefold, from 4136 in 2012 to 1383 in 2020. The incidence of tuberculosis among men was higher than among women. There was an increase in strains’ resistance to antimycobacterial drugs in both newly diagnosed patients, from 4.4% in 2012 to 6.1% in 2020, and previously treated patients, from 11.7% to 12.3%. Four-year resistance increased to 1% and 2.1%, respectively. The spoligotype SIT1 was the most abundant among the resistant strains (17%), and SIT53 (13.9%) was the most common among susceptible strains.

## 1. Introduction

Tuberculosis (TB) is an infectious disease caused by mycobacteria of the *Mycobacterium tuberculosis* complex. Until the outbreak of the coronavirus pandemic (COVID-19), TB was the leading cause of death from a single infectious agent, ranking higher than HIV (AIDS). Unfortunately, the COVID-19 pandemic set back years of progress in the fight against tuberculosis, causing a global decline in the number of newly diagnosed and reported TB patients. The number of newly diagnosed TB patients has declined by approximately 20%, to the level recorded in 2012 (from 7.1 million in 2019 to 5.8 million in 2020) [1], representing a setback of at least 5 to 8 years in the fight against TB due to the COVID-19 pandemic. Limited access to diagnosis and antimycobacterial treatment has also resulted in an increase in deaths. In 2020, for the first time in more than a decade, an increase of more than 100,000 deaths was recorded, reaching 1.3 million [1]. The consequences of not having access to basic TB diagnostic and treatment services are expected to rise in future years. Additionally, the number of patients treated for drug-resistant TB decreased by 15%, from 177,000 in 2019 to 150,000 in 2020 [1]. Poland is one of the EU member states demonstrating low TB incidence rates (13.9 cases per 100,000 in 2019). In 2010, a decline in TB incidence to <20 per 100,000 population was first reported, and the downward trend has continued since then [2]. It is worth mentioning that the incidence rates of TB in post-war Poland were extremely high, at >290 per 100,000; thus, a substantial percentage of the population was infected with mycobacterium tuberculosis, and the immediate eradication of the disease was not possible. The radical decrease of epidemiological indicators in tuberculosis is certainly one of the greatest successes in Polish medicine [3]. In 2019, 5321 cases of tuberculosis were registered in Poland, which is 166 cases of tuberculosis fewer than in the previous year and 2188 cases fewer than in 2010. The incidence of all forms of TB was 13.9 in 2019, down 2.8% from 2018 and down 29.4% from 2010, when it was 19.7 [4]. In 2020, in the midst of the COVID-19 pandemic, 3388 TB cases were registered, which was 1993 fewer than the previous year. The incidence of all forms of TB was 8.8 in 2020, a decrease of 36.7% compared to 2019 [2]. Despite significant improvement in the epidemiological situation, the prevalence of tuberculosis is slightly higher than the figures in other Western European countries: for example, 8.1 per 100,000 inhabitants in Germany, 7.7 in France, or 8.2 in Sweden [5]. Among the methods of diagnosing TB, microbiological methods are the gold standard and are crucial, as they allow for correct diagnosis and rapid initiation of treatment with the most effective regimen. Most clinical features of TB have low specificity, which can lead to misdiagnosis and unnecessary treatment [6].

The drug-resistant form of TB, in particular multidrug-resistant (MDR), pre-extensively drug-resistant, and extensively drug-resistant (XDR), constitutes a recent health concern and challenge for TB-control programmes worldwide. Monitoring the drug resistance of *Mycobacterium tuberculosis* strains to antituberculosis drugs is an important aspect of TB surveillance and is helpful in identifying the predominant MDR-TB strains and in indicating the quality of TB control in a country. Early detection and diagnosis of patients prevents transmission of drug-resistant strains in the environment [7]. The priority should be to restore access to essential TB services and increase spending on diagnostics, treatment, and prevention so that detection and treatment levels can return to at least those of 2019 [1].

The aim of this study was to investigate the effect of the COVID-19 pandemic on the diagnosis of tuberculosis in Poland and the patterns of resistance to basic antimycobacterial drugs shown by *M. tuberculosis* strains isolated in both newly diagnosed and previously treated patients. The epidemiological analysis of the 2020 strains was a cyclic study (conducted every 4 years) at the National Reference Laboratory for Mycobacteria at the Institute of Tuberculosis and Lung Diseases in Warsaw, according to the WHO protocol. The results were compared not only with data obtained in 2012 and 2016 but also with data reported to the National Tuberculosis Registry (NTR) [2,8,9]. Molecular analysis was performed on 20% of the strains susceptible to the four primary antimycobacterial drugs and all of the resistant strains in order to determine the frequency of specific molecular patterns of shared international type (SIT) spoligotype in the group of strains susceptible and resistant to antimycobacterial drugs.

## 2. Materials and Methods

The study was retrospective and prospective, based on the results of routinely performed microbiological tests at Mycobacterium Tuberculosis Laboratories in Poland. *Mycobacterium tuberculosis* complex strains cultured in regional laboratories were sent to the National Reference Laboratory for Mycobacteria at the Institute of Tuberculosis and Lung Diseases in Warsaw, together with a questionnaire containing information on the strain (the specimen from which it was cultured, basic identification tests, and drug resistance determined in the field laboratory) and data on the patient (sex, age, form of tuberculosis, and previous antimycobacterial treatment). The isolation was performed using Löwenstein–Jensen medium or BD Bactec MGIT system (Becton Dickinson Microbiology Systems, Cockeysville, MD, USA), with species identification based on niacin tests, the use of BD MGIT TBc identification test (TBc ID), and nucleic acid amplification test (NAAT). Drug susceptibility testing used the proportion method in Löwenstein–Jensen medium or using the BD Bactec MGIT 960 system.

The total number of *Mycobacterium tuberculosis* strains analysed in 2020 was 1383. The results were compared to studies conducted in 2012 and 2016. The programmes in these years followed the same WHO protocol and included approximately 9000 TB patients. Molecular analysis entailed spoligotyping for 20% (252) of the strains susceptible to 4 antimycobacterial drugs and for all (82) of the strains resistant to at least 1 drug from 2020. This is a pilot study. In the next stages, spoligotyping will be performed for the remaining 80% of strains sensitive to antituberculosis drugs.

### Method for Spacer Oligonucleotide Typing (Spoligotyping)

Spoligotyping was performed by amplifying direct repeat regions in the genome of *M. tuberculosis* complex with the primers DRa and DRb and an available spoligotyping kit (Ocimum Biosolutions, Hyderabad, India) according to the protocol [10]. The amplified products were then hybridised to a membrane pre-coated with spacer oligonucleotides that characterise the spacer region of the identified sequence. After incubation with streptavidin-peroxidase and enhanced chemiluminescence detection, the presence of spacers was visualised on X-ray films as black squares [11,12]. *M. tuberculosis* H37Rv was used as a positive control. The resulting spoligotypes were compared to the patterns registered in the SITVIT2 international database, available at http://www.pasteur-guadeloupe.fr:8081/SITVIT2 (accessed on 1 February 2022).

Among the 96 drug-resistant strains analysed, hybridisation patterns were obtained for 82. The strains were cultured from 64 (78%) Polish citizens and 18 (22%) foreigners living in Poland (from Ukraine, Georgia, Moldova, Vietnam, Nepal, and the Philippines). As with the resistant strains, genotyping was performed by spoligotyping. Hybridisation patterns were obtained for 252 out of 1287 sensitive strains. Strains were cultured from 201 (79.8%) Polish citizens and 51 (20.2%) foreigners living in Poland (from Ukraine, India, Nepal, Bangladesh, South Africa, and other countries).

## 3. Results

### 3.1. Sex and Age of Tuberculosis Patients in 2020

Among the 1383 patients from whom mycobacteria belonging to *Mycobacterium tuberculosis* complex were cultured, the most numerous group included men aged 55–64 years (26.03%). Among women, most patients were over 65 years old (24.32%). The incidence of TB among men was higher than among women. There were 1087 cases registered in men and 296 cases in women. There were five children (0.36%) in the study group: two boys and three girls. They belonged exclusively to the group of newly diagnosed patients. Three of them were under 5 years of age (Table 1).

Comparing the sex of TB patients in our three original studies—from 2012, 2016, and 2020—with data from the NTR in Poland, we found that the percentages of female and male patients in all groups were similar. Men were more likely to contract the disease in all three studies. In the 2012 and 2016 patient groups, the female-to-male ratio averaged 1:2.5, while it reached 1:3.7 in the 2020 patient group. Similarly, data from the NTR show that in 2020, the proportion of male patients increased by about 5 p.p. over previous years, reaching 76.3% (Table 2).

Data on patients with incomplete clinical information on the course of treatment, preventing patients from being classified as newly diagnosed or previously treated, were excluded from the analysis. The percentages of newly diagnosed and previously treated cases did not change significantly among the three studies by the authors, amounting to 87% and 13%, respectively. In contrast, in absolute numbers, there was a significant decrease in patients reported in 2020 compared to previous years. The number of strains submitted dropped threefold, from 4136 in 2012 to 1383 in 2020. The NTR shows that reported TB cases halved, from 5070 in 2012 to 2655 in 2020 (Table 3).

### 3.2. Analysis of Primary and Acquired Drug Resistance in Authors’ 2020 Study

Newly diagnosed patients (1174) accounted for 84.9% of the cases, with 1102 of them (93.9%) isolated strains that were sensitive to all tested drugs. Resistant mycobacteria were isolated from 72 patients (6.13%). There were 46 (3.9%) mono-resistant strains, most commonly resistant to isoniazid (I) and streptomycin (S), at 25 (2.13%) and 19 (1.62%), respectively. Nine patients showed resistance to two drugs, and five patients to three drugs. Resistance to all four primary drugs was observed in 12 patients. Multiple-drug resistance, i.e., resistance to at least isoniazid (I) and rifampicin (R), was found in 19 patients (1.6%). Among MDR strains, resistance to the following four antimycobacterial drugs prevailed (SIRE): streptomycin, isoniazid, rifampicin (R), and ethambutol. Three drug-resistant (SIR) and two drug-resistant (IR) strains were isolated from five patients (Table 4).

Among the 187 patients (13.5%) previously treated with antimycobacterial drugs, 164 (87.7%) isolated strains were sensitive to the primary drugs, and 23 (12.3%) isolated strains were resistant to at least 1 drug. Twelve patients (6.42%) were infected with mycobacteria resistant to a single drug, and four patients each had a strain with mono-resistance to streptomycin, isoniazid, or rifampicin. MDR-TB drug resistance was found in 10 cases (5.3%). Among the MDR strains, four-drug resistance (SIRE) was the most common, manifesting in four patients. Resistance to three drugs was found in four patients—three SIR and one IRE—while two patients showed resistance to two drugs (IR). Only one strain showed resistance with a different phenotype, that of the SIE type (Table 4).

Information on history of TB disease and treatment was not obtained for 21 of the patients (1.5%).

**Figure 1:**
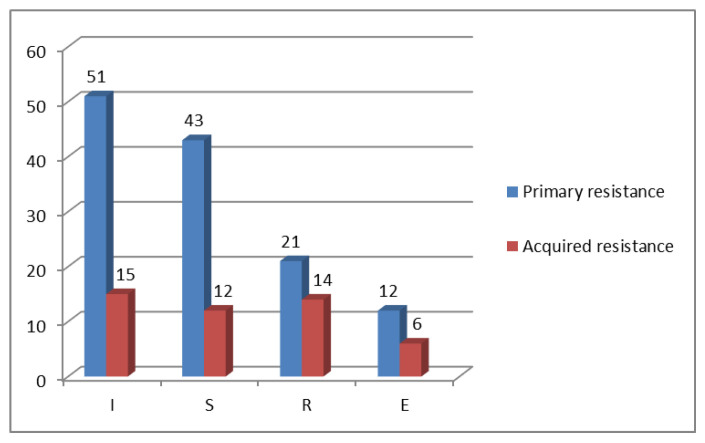
Total resistance to I, S, R, and E in *M. tuberculosis* strains isolated from new (primary resistance) and previously treated patients (acquired resistance), 2020.

The analysis of total drug resistance (the proportion of individual drugs in all resistance patterns) shows that isoniazid resistance prevailed in the groups of newly diagnosed and previously treated patients, with 51 (70.8%) and 15 (65.2%), respectively; ethambutol resistance was the least common, with 12 (16.7%) and 6 (26.1%), respectively. There were 43 (59.7%) and 12 (52.2%) cases, respectively, of streptomycin resistance. Resistance to rifampicin was found in 21 (29.2%) and 14 (60.9%) cases, respectively (Figure 1).

### 3.3. Molecular Analysis of Mycobacterium Tuberculosis Strains Resistant to at Least 1 Drug (Compared to 20% of Sensitive Strains), Poland 2020

Among the 82 hybridisation patterns (drug-resistant strains), the SITVIT2 international database identified spoligotypes most commonly belonging to the Beijing 22 (26.8%) and T 22 (26.8%) families, followed by Haarlem 18 (21.9%), URAL 6 (7.3%), LAM 5 (6.1%), and EAI 2 (2.4%) families. Twenty-four individual (unique) spoligotypes were identified. The following spoligotypes were the most abundant among resistant strains cultured in 2020: SIT1 14 (17%), SIT53 8 (9.7%), SIT265 8 (9.7%), and SIT139 6 (7.3%). Seven patterns had no counterparts in the database, with a 15-digit octagonal number only. Two isolates had the same pattern: 777737607420771 (Table 5).

In order to determine or exclude differences in the molecular patterns of drug-sensitive and drug-resistant strains, 20% of the strains were randomly selected among the sensitive strains isolated from patients in 2020. The SITVIT2 international database identified spoligotypes most commonly belonging to the T 77 (30.5%), Haarlem 64 (25.4%), URAL 17 (6.7%), and Beijing 15 (5.9%) families, followed by CAS 10 (4%), LAM 9 (3.6%), EAI 3 (1.2%), and X 3 (1.2%) and S 2 (1.2%). Forty-eight unique spoligotypes were identified. Spoligotypes were the most abundant among the susceptible strains grown in 2020: SIT53 35 (13.9%), SIT50 21 (8.3%), SIT47 17 (6.7%), and SIT1 14 (5.5%). Another 52 patterns were not found in the database. The most common among them was the pattern 770000777660731, which represented 10 strains of *Mycobacterium tuberculosis* (Table 6).

## 4. Discussion

As a consequence of the COVID-19 pandemic, the WHO predicts that the epidemiological situation of tuberculosis will deteriorate worldwide [13]. The pandemic caused significant changes in the functioning of health care systems, other important epidemiological problems were neglected, and the diagnosis of numerous infectious diseases, including tuberculosis, became less important. This may result in weaker national TB programmes [14] and increased TB incidence in the near future [15]. There has been a downward trend in TB incidence rates in Poland since 1957. In 2020, the incidence of TB was 8.8, significantly lower than in 2018 and 2019 (14.3 vs. 13.9) [2]. Unfortunately, the low rate in 2020 was a result of the COVID-19 pandemic. The ERLTB-Net-2 network of European reference mycobacterial laboratories published a report on the impact of the COVID-19 pandemic on TB laboratory services in Europe. They found that the most severe disruption of TB NRL services occurred at the beginning of the pandemic and coincided with a significant decrease in the number of samples received, by about 30% [16]. A similar analysis conducted by the National Reference Laboratory for Mycobacteria in Poland found that the number of TB tests decreased by as much as 45% during a single year of the pandemic [2]. A study by Migliori et al. in 33 centres from 16 countries [17] assessed patient attendance at TB health care units by comparing data from 4 months of the COVID-19 pandemic (January–April 2020) within the same period in 2019. Most centres reported a decrease in the number of newly diagnosed TB cases and the total number of outpatient visits for active disease. In some centres, medical staff working with TB patients have been seconded to work with COVID-19 patients. In addition, the fewer clinic visits were due to patients’ fear of COVID-19 exposure or difficulty accessing medical services [18].

As our comparative analysis has shown, the breakdown of the sex and age of TB patients in Poland has remained unchanged for years. The highest incidence of TB is among Poles over the age of 44. It is primarily men who get sick. Three times more men die from TB than women in Poland [6]. In 2020, men between the ages of 45 and 64 were also the largest group of patients (26%). Among women, most patients were over 65 years old (24%). Children under 14 years of age accounted for only 0.36%. The incidence of tuberculosis in the paediatric population mirrors the epidemiological situation of tuberculosis among adults. The new incidence of tuberculosis in children indicates that mycobacteria are being transmitted in the environment and that the disease is not completely controlled [19].

When comparing the results obtained in the three consecutive studies, secondary drug resistance was found to be statistically significantly more frequent than primary drug resistance. At the same time, the number of patients excreting mycobacteria with MDR resistance was more common among the previously treated patients than in the newly diagnosed ones. The proportion of patients excreting MDR-resistant mycobacteria ranged from 0.6% in 2012 to 5.3% in 2020 (Table 7).

**Figure 2:**
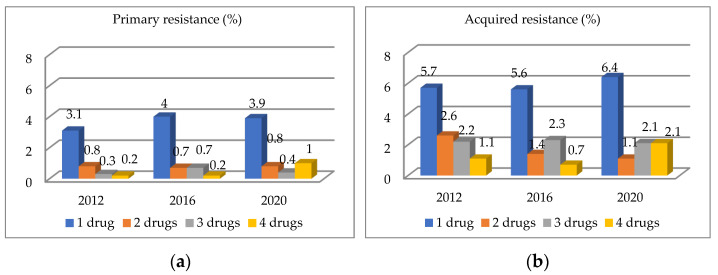
Resistance to one or more drugs among newly diagnosed (**a**) and previously treated (**b**) patients in Poland in 2012, 2016, and 2022.

In the three studies from 2012, 2016, and 2020, the highest proportion of newly diagnosed patients excreted mycobacteria that was resistant to a single drug (3.1%, 4%, and 3.9%, respectively). Patients with tuberculosis resistant to two drugs accounted for 0.8% of all newly diagnosed patients. The highest percentage of three-drug resistance was recorded in 2016 at 0.7%, while in 2012 and 2020, it was 0.3% and 0.4%, respectively. In the newly diagnosed patients in the 2012 and 2016 studies, four-drug resistance was found to be 0.2%, whereas this group constituted 1% in 2020 (Figure 2a).

Among the previously treated patients, as with the group of newly diagnosed patients, the greatest number of them excreted mycobacteria that was resistant to a single drug, about 6%. The number of strains resistant to two drugs decreased steadily from 2.6% in 2012 to 1.1% in 2020. The number of mycobacteria resistant to three drugs remained stable at 2.2%. The percentage of TB patients resistant to the four SIRE drugs also increased to 2.1% in 2020 (Figure 2b).

This may be one of the effects of the COVID-19 pandemic, resulting in fewer available records and less monitoring of TB treatment in Poland.

Tracking the rise of mycobacterial resistance and implementing prevention methods is an important method for surveillance of the spread of tuberculosis. *Mycobacterium* drug resistance is the result of insufficient inhibition of mycobacteria growth by drugs’ suboptimal concentrations caused by the administration of inappropriate drug combinations at inappropriate doses, for example, ref. [6]. Multi-drug resistant MTB strains are a growing health problem and a major challenge for TB control programmes. Knowledge of the prevalence of resistant strains in a population provides essential information about the epidemiology of the disease in a country. Most of the TB data obtained for 2020 in Poland are significantly lower than in previous years, demonstrating the limited availability of TB diagnosis and treatment during the COVID-19 pandemic. It is no coincidence that the areas of the world projected to be most affected by the social and economic consequences of COVID-19 are also the areas with the highest TB burden [20]. This is because TB is both a social and infectious disease. Poorer, malnourished people living in densely populated areas are more vulnerable to TB, and TB exacerbates poverty by increasing costs, reducing income and being associated with stigmatisation and discrimination [21,22,23,24,25].

Molecular analysis of the incidence of major SITs in Poland in 2020 revealed 35 different spoligotypes and six patterns not registered in the global SITVIT2 database among strains resistant to at least one antimycobacterial drug. It is noteworthy that in the group of patients excreting drug-resistant mycobacteria, in addition to the T family, which prevails in the European population (26.8%), the same percentage of strains was also registered for the Beijing family. The *Mycobacterium tuberculosis* genotype with the canonical spoligotype SIT1 was first described in 1995 and is now the predominant strain among TB patients in many Asian countries though it is increasingly being identified in all seven geographical areas of the world [26]. In Europe, Beijing strains have emerged as endemic and dominant genotypes in countries of the former Soviet Union, often in association with drug resistance [27,28,29,30]. Due to human migration and mobility, significant changes in the breakdown of MTB strains have been observed in other European countries, such as Ireland and Germany [31,32]. In Poland, non-Beijing genotypes were most common among drug-resistant strains until 2016, whereas since 2017, the Beijing genotype has prevailed. This is due to the fact that until recently, Beijing TB was identified in Poland mainly in foreigners from Eastern Europe and Asia, with a rise in cases identified among Poles since 2017. Considering all this, TB-control programmes should also use molecular epidemiology to track the transmission of high-risk strains and the diversity of TB in a given area. In European countries with a low incidence of tuberculosis, the intercontinental migration of people for recreation, work, or because of armed conflicts can dramatically change the socio-epidemiological situation. Among sensitive strains, there were 73 spoligotypes and 30 patterns that were not registered in the database. Unregistered patterns accounted for 11.9% of the 252 strains tested. Orphan spoligotypes represent patterns and were identified for the first time in a group of Polish patients in this study. These may indicate recent and/or sporadic TB transmission in the study area [12,25]. The SITVIT2 database shows that this origin is more common among susceptible strains than drug-resistant strains in Poland. Among MTB strains sensitive to antimycobacterial drugs, SIT53 was the most common spoligotype in Poland. Large-scale migration from countries with high TB incidence rates can lead to unexpected changes in epidemiological indicators through the transmission of MTBC strains not previously recorded in the population. Therefore, it seems that the molecular identification of circulating clades is extremely important in controlling the epidemiological situation of tuberculosis worldwide [12].

## Figures and Tables

**Table 1:** Tuberculosis incidence in Poland in 2020, by age and sex.

Sex	Total	Number of Cases in Age Groups (Years)						
		0–14	15–24	25–34	35–44	45–54	55–64	65+
**Male**	1087 (8 no data)	2	38	108	205	259	283	184
		0.18%	3.49%	9.93%	18.85%	23.82%	26.03%	16.92%
**Female**	296 (1 no data)	3	25	36	58	54	47	72
		1.01%	8.44%	12.16%	19.59%	18.24%	15.88%	24.32%
**Total**	1383 (9 no data)	5	63	144	263	313	330	256
		0.36%	4.55%	10.41%	19.02%	22.63%	23.86%	18.51%

**Table 2:** Comparison of the number of culture-confirmed tuberculosis cases between the authors’ studies and data from the NTR in Poland in 2012, 2016, and 2020, by sex.

	Year	Total	Female	Male
Own studies	2012	4136	1185 (28.7%)	2951 (71.3%)
	2016	3591	990 (27.6%)	2601 (72.4%)
	2020	1383	296 (21.4%)	1087 (78.6%)
Registered in the NTR	2012	5070	1509 (29.8%)	3561 (70.2%)
	2016	4619	1311 (28.4%)	3308 (71.6%)
	2020	2655	630 (23.7%)	2025 (76.3%)

**Table 3:** Comparison of data on new cases and relapse tuberculosis cases between the authors’ studies and data from the National Tuberculosis Registry (NTR) in Poland in 2012, 2016, and 2020.

	Year	Total	New TB Cases	Relapse TB Cases
Own studies (n = 8938)	2012	4136	3596 (87%)	540 (13%)
	2016	3441	3012 (87.5%)	429 (12.5%)
	2020	1361	1174 (86.3%)	187 (13.7%)
Registered in the NTR (n = 12,344)	2012	5070	4475 (88.3%)	595 (11.7%)
	2016	4619	4106 (89%)	513 (11%)
	2020	2655	2268 (85.4%)	387 (14.6%)

**Table 4:** Resistance patterns of *Mycobacterium tuberculosis* strains isolated from newly diagnosed patients (primary drug resistance) and from previously treated patients (acquired drug resistance) in Poland in 2020.

	Primary Drug Resistance	Acquired Drug Resistance
	% (n)	% (n)
**Total**	100 (1174)	100 (187)
**Sensitive**	93.87 (1102)	87.7 (164)
**Resistant**	6.13 (72)	12.3 (23)
**1 drug**	3.92 (46)	6.42 (12)
**S**	1.62 (19)	2.14 (4)
**I**	2.13 (25)	2.14 (4)
**R**	0.17 (2)	2.14 (4)
**E**	0 (0)	0 (0)
**I + R + other**	1.62 (19)	5.35 (10)
**IR**	0.17 (2)	1.07 (2)
**IRS**	042 (5)	1.6 (3)
**IRE**	0 (0)	0.53 (1)
**IRES**	1.02 (12)	2.14 (4)
**I + other**	0.6 (7)	0.53 (1)
**IS**	0.6 (7)	0 (0)
**IES**	0 (0)	0.53 (1)

**Table 5:** Prevalence of the most common spoligotypes of resistant strains of *Mycobacterium tuberculosis* in Poland in 2020 (n = 82).

LSP/SNP-Based	Spoligotype Family	Lineage	SIT	Isolates in Study
**East Asian**	Beijing		1	14
			265	8
**Euro-American**	T	T1	53	8
		T4	139	6
		T5	44	2
		T1	558	2
		unique		4
	Haarlem	H1	47	3
		H3	50	2
		H3	36	2
		H4	262	2
		unique		9
	LAM	LAM9	42	2
		unique		3
	URAL	unique		6
**Indo-Oceanic**	EAI	unique		2
***Unregistered***				*7*

**Table 6:** Prevalence of the most common spoligotypes of sensitive strains of *Mycobacterium tuberculosis* in Poland in 2020 (n = 252).

LSP/SNP-Based	Spoligotype Family	Lineage	SIT	Isolates in Study
**East Asian**	Beijing		1	14
		unique		1
**Euro-American**	T	T1	53	35
		T5	44	6
		T4	40	3
		T4	139	3
		T5	254	3
		T1	2	3
		T3	37	3
		T1	462	2
		T5	68	2
		T1	191	2
		unique		15
	Haarlem	H3	50	21
		H1	47	17
		H3	36	5
		H1	382	3
		H4	262	3
		H4	35	3
		H1	51	2
		unique		10
	LAM	LAM9	42	5
		unique		4
	URAL		46	3
			237	3
			124	2
			602	2
		unique		7
	S		34	2
	X	unique		3
**East-African-Indian**	CAS	CAS1	26	5
		unique		5
**Indo-Oceanic**	EAI	unique		3
***Unregistered***				*52*

**Table 7:** Comparison of results in newly diagnosed and previously treated patients in Poland 2012, 2016, and 2020.

Year	TB Primary/Acquired	Number of Patients Studied	Number of Patients with Resistant Mycobacteria (%)	Number of Patients with MDR (%)
**2012**	P	3596	157 (4.4%)	20 (0.6%)
	W	540	63 (11.7%)	24 (4.4%)
**2016**	P	3012	168 (5.6%)	32 (1.1%)
	W	429	43 (10%)	18 (4.2%)
**2020**	P	1174	72 (6.1%)	19 (1.6%)
	W	187	23 (12.3%)	10 (5.3%)

## Data Availability

Data supporting reported results can be found in source data collected in National Tuberculosis and Lung Diseases Research Institute.
